# Adaptive Bayesian Iterative Transmission Reconstruction for Attenuation Correction in Myocardial Perfusion Imaging with SPECT/Slow-Rotation Low-Output CT Systems

**DOI:** 10.1155/2007/18709

**Published:** 2007-02-15

**Authors:** Ji Chen, Ernest V. Garcia, Russell D. Folks, Aharon Peretz, James R. Galt

**Affiliations:** ^1^Department of Radiology, School of Medicine, Emory University, 1364 Clifton Road, Atlanta, GA 30322, USA; ^2^GE Healthcare, 39120 Haifa, Israel

## Abstract

*Objectives*. SPECT/slow-rotation low-output CT systems can produce streak artifacts in filtered backprojection (FBP) attenuation maps, impacting attenuation correction (AC) in myocardial perfusion imaging. This paper presents an adaptive Bayesian iterative transmission reconstruction (ABITR) algorithm for more accurate AC. *Methods*. In each iteration, ABITR calculated a three-dimensional prior containing the pixels with attenuation coefficients similar to water, then used it to encourage these pixels to the water value. ABITR was tested with a cardiac phantom and 4 normal patients acquired by a GE Millennium VG/Hawkeye system. *Results*. FBP AC and ABITR AC produced similar phantom results. For the patients, streak artifacts were observed in the FBP and ordered-subsets expectation-maximization (OSEM) maps but not in the ABITR maps, and ABITR AC produced more uniform images than FBP AC and OSEM AC. *Conclusion*. ABITR can improve the quality of the attenuation map, producing more uniform images for normal studies.

## 1. INTRODUCTION

Attenuation correction (AC) has undergone extensive clinical investigation [1–4] and now is a recommended technique for improving the quality of myocardial perfusion imaging (MPI) with single-photon emission computed tomography (SPECT) [5]. Since the attenuating material in a patient's thorax is too varied to meet the constant attenuation coefficient approximation made in both Sorenson's and Chang's methods [6, 7], transmission imaging is required to obtain patient-specific attenuation maps for accurate AC in MPI [8–11]. It has been pointed out that high-quality transmission scans and sufficient transmission counts with low crosstalk from the emission radionuclide are essential to reduce the propagation of noise and error into the attenuation-corrected emission images [5].

**Figure 1:**
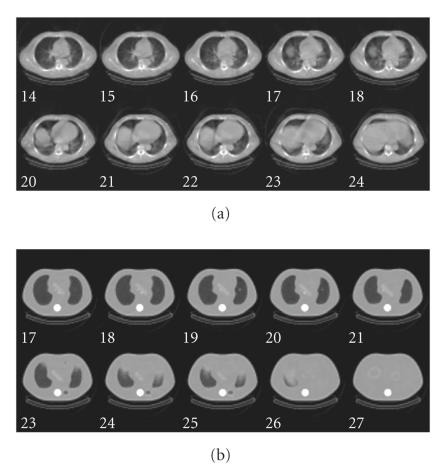
Example Hawkeye CT attenuation maps of (a) patient and (b) phantom.

Recently, hybrid X-ray CT and SPECT systems became available for MPI. Among the commercial SPECT/CT systems, Millennium VG/Hawkeye (GE Healthcare Technologies, Milwaukee, Wis) produces the lowest cost and dose to the patient, since this camera uses a single-slice, slow-rotation, low-output CT scanner. AC with these systems has shown to improve sensitivity, specificity, and predictive accuracy in detection of coronary artery disease [12]. Compared to the conventional radionuclide attenuation maps, the Hawkeye CT attenuation maps have higher resolution and contrast and do not have the crosstalk and low-count issues. With phantom models, it has been shown that Hawkeye AC is, to some extent, superior to AC given by the SPECT systems with radionuclide transmission sources [13]. However, in clinical patients streak artifacts are often observed in the attenuation maps produced by filtered backprojection (FBP) of the slow-rotation low-output CT scans (shown in Figure 1). These artifacts are produced by inconsistencies in the CT sinogram such as those caused by respiratory motion during the CT acquisition. It has become a serious concern that these streak artifacts may degrade the accuracy of AC, and essentially raise the probability of false-positive cases in clinical practice.

Bayesian techniques have been introduced to transmission reconstruction for AC using radionuclide transmission imaging [14, 15]. It has been validated that Bayesian techniques can better handle the low-count issue associated with radionuclide transmission imaging than FBP, so as to enhance the quality of the reconstructed attenuation maps and improve the accuracy of AC [16, 17]. Although transmission noise is not an issue with the Hawkeye CT data, Bayesian technique may be useful in transmission reconstruction of the Hawkeye CT data for reducing the streak artifacts and improving the quality of the attenuation maps. Based on this hypothesis, this study developed an adaptive Bayesian iterative transmission reconstruction (ABITR) algorithm in order to remove the streak artifacts in the Hawkeye CT attenuation maps for more accurate AC.

## 2. MATERIALS AND METHODS

### 2.1. Phantom and patient studies

**Figure 2:**
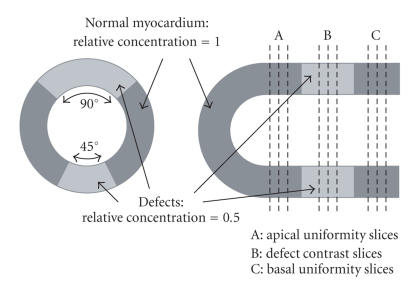
The cardiac insert configuration of the phantom.

A data spectrum anthropomorphic torso phantom with cardiac, liver, lung, and spine components was used in this study. The cardiac insert consisted of a plastic chamber simulating the left ventricular chamber surrounded by a 1-cm-thick plastic chamber simulating the myocardium. Two smaller fillable chambers (90° and 45°, resp.), 1 cm thick and 2 cm long, were used to simulate hypoperfused defects (illustrated in Figure 2). The normal myocardium and defects were filled with 222 kBq/mL (6 *μ*Ci/mL) and 111 kBq/mL (3 *μ*Ci/mL) of Tc-99m, respectively, simulating typical patient uptakes of Tc-99m sestamibi or Tc-99m tetrofosmin. The liver and background were filled with 11.1 kBq/mL (0.3 *μ*Ci/mL) of Tc-99m.

This phantom was imaged four times with a GE Millennium VG/Hawkeye system using the following acquisition parameters:

1. low-energy high-resolution collimators;
2. 20% photopeak window centered at 140 keV;
3. 12% scatter window centered at 118 keV;
4. 180° acquisition, noncircular orbit;
5. 60 projections, 30 seconds per projection.

The raw CT sinograms were preprocessed for crosstalk correction, nonlinearity correction, air and monitor normalization, and ring artifact correction by means of the manufacturer-provided tools. The preprocessed CT sinograms were rebinned from fan-beam geometry to parallel-beam geometry. In the rebinning, the pixel size of the CT sinograms was changed to the pixel size of the emission images. The rebinned sinogram was then submitted to ordered-subsets expectation-maximization (OSEM) (standard iterative reconstruction, without the Bayesian prior) and ABITR. Standard FBP (available on the system) was also used to reconstruct the attenuation maps for comparison. The FBP reconstruction was performed before the rebinning. The FBP attenuation maps were then rebinned to the same pixel size as the OSEM and ABITR attenuation maps.

Compton window subtraction [18] was performed on the tomographic projections as

(1) *P*_scatter-compensated_ = *P*_photopeak_ − *k* × *P*_scatter_,

where *P*_scatter-compensated_ is the scatter-compensated tomographic projection, *P*_photopeak_ and *P*_scatter_ are tomographic projections in the photopeak window and scatter window, respectively, *k* is the scatter compensation scaling factor (the manufacturer-recommended value 1 was used). The scatter-compensated tomographic projections were submitted to emission reconstruction with AC. OSEM with 3 iterations and 6 subsets was used for the emission reconstruction. The reconstructed images then underwent three-dimensional (3D) Butterworth postfiltering (critical frequency of 0.4 cycles/cm and power of 10) followed by cardiac reorientation. Attenuation corrected short-axis images using the FBP, OSEM, and ABITR attenuation maps were then submitted to quantitative comparison and statistical analysis.

Four normal patients were used to compare OSEM AC and ABITR AC to FBP AC. These patients underwent a standard stress Tc-99m sestamibi rest Tl-201 dual-isotope protocol using the GE Millennium VG/Hawkeye system with the same acquisition parameters as those in the phantom experiments. Short-axis images given by FBP AC, OSEM AC, and ABITR AC were obtained using the same reconstruction parameters as above and then submitted to quantitative comparison and statistical analysis.

### 2.2. Adaptive Bayesian iterative transmission reconstruction

**Figure 3:**
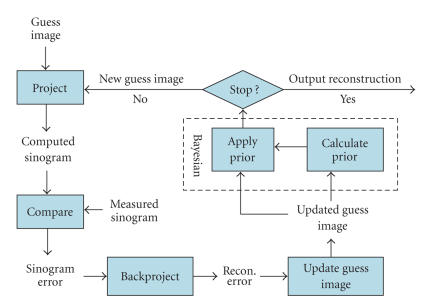
Illustration of the adaptive Bayesian iterative transmission reconstruction (ABITR) algorithm.

ABITR is illustrated in Figure 3. The input of ABITR is a uniform image as initial guess image and a measured CT sinogram after preprocessing. A guess sinogram was generated from the guess image and then compared to the measured sinogram. The error of this comparison was backprojected, and then used to update the guess image. A 3D Bayesian prior image was calculated by image segmentation of the updated guess image. The image segmentation identified an object image which contained the pixels whose values were close to the water attenuation coefficients (±25%). Morphological processing was then applied to the object image to improve the results from image segmentation [19]. Morphological opening (removing small objects from an image while preserving the shape and size of large objects in the image) and closing (filling in small gaps inside large objects and smoothing the outer edges of large objects) were used to reduce the impact on image segmentation from the streak artifacts in the guess image. After the prior image was obtained, the pixels inside the object was updated using the following equation:

(2) Pixel_new_ = (1 − *b*) × Pixel_old_ + *b* × *μ*_water_,

where Pixel_old_ and Pixel_new_ were the original and updated values of the pixels in the guess image that were inside the object, *b* is a factor controlling the strength of the Bayesian prior (with *b* equal to 0, the ABITR algorithm becomes a standard OSEM algorithm), and *μ*_water_ is the water attenuation coefficient, 0.153 cm^−1^ for 140 keV photopeak.

The guess image updated with the Bayesian prior was then submitted to forward projection to start a new iteration. ABITR stopped at a preset iteration number (30 iterations used in this study).

ABITR was performed on a PC with Pentium IV 2.4 GHz CPU and 512 Mb RAM. It was a totally automatic process and took less than 2 minutes to reconstruct a map with a 64 × 64 matrix and 25 slices.

### 2.3. Quantitative comparison and statistical analysis

Defect contrast and normal short-axis uniformity were used to compare the ABITR AC with the FBP AC in the phantom studies. Maximal-count circumferential profile (MCCP), extracted from a short-axis slice, was used to calculate the contrast and uniformity [20]. Three short-axis slices cutting through the two defects, as shown in Figure 2, were selected to calculate their defect contrasts, and their mean value represented the defect contract of that region. The defect contrast was defined as

(3) $$\text{contrast}=\frac{\left[\max\left(\text{MCCP}\right)-\min\left(\text{MCCP}\right)\right]}{\max\left(\text{MCCP}\right)}$$,

where max(MCCP) and min(MCCP) were maximal and minimal counts in the MCCP, respectively. The MCCPs extracted from three short-axis slices cutting through regions of homogeneous tracer distribution towards the base and towards the apex were selected to calculate their normal short-axis uniformities (shown in Figure 2), and their mean value represented the basal and apical uniformities, respectively. The uniformity was defined as

(4) $$\text{uniformity}=\frac{\text{stdev}\left(\text{MCCP}\right)}{\text{mean}\left(\text{MCCP}\right)}$$,

where stdev(MCCP) and mean(MCCP) were the standard deviation and mean of the counts in the MCCP, respectively. The optimal defect contrast should be close to 50% and optimal uniformity should be close to 0.

Short-axis uniformity, the same quantitative index as above, was used to compare the OSEM AC and ABITR AC to the FBP AC in the patient studies for the apical, middle, and basal regions.

Paired *t*-test was used to compare the contrast and uniformity in the phantom studies and the uniformity in the patient studies.

## 3. RESULTS

**Figure 4:**
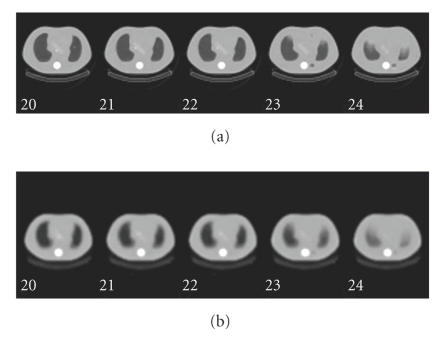
Phantom attenuation maps: (a) the FBP map (b) the ABITR map.

**Figure 5:**
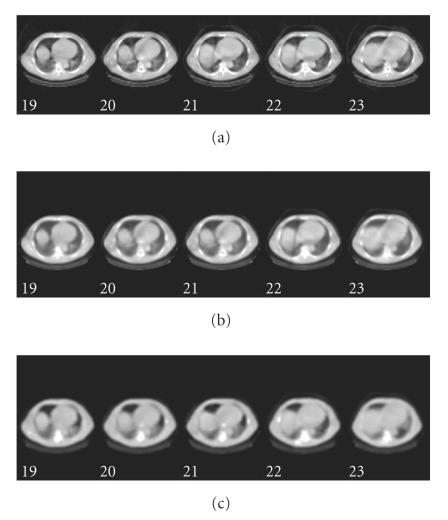
Patient (a) FBP, (b) OSEM, and (c) ABITR attenuation maps.

Figure 4 shows the FBP and ABITR attenuation maps in phantom. Both maps looked similar. Figure 5 shows the FBP, OSEM, and ABITR attenuation maps in patient. Both FBP and OSEM maps had streak artifacts, not observed in the ABITR map.

The uniformity and defect contrast analyses of the phantom studies are shown in Table 1. No statistically significant difference was found between FBP AC and ABITR AC in the phantom studies. Table 2 shows the short-axis uniformity analysis of the patient studies. The ABITR AC images were barely significantly (*P* = .0547) more uniform at the basal region compared to the FBP AC images for the normal patient studies. No significant differences were obtained between the OSEM AC and FBP AC images, indicating that the Bayesian process played more important role in improving the quality of the attenuation map than iterative reconstruction.

**Figure 6:**
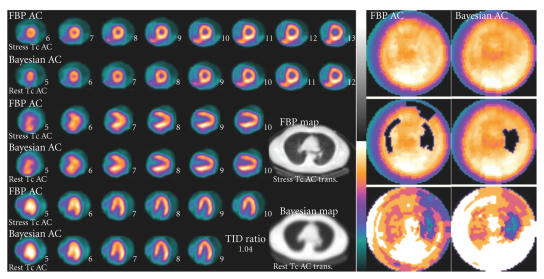
Images of a normal subject. FBP (top) and Bayesian (bottom) attenuation maps and AC images are shown on the left panel. FBP and Bayesian polar maps (three for each, shown as a column) are shown on the right panel. For each column, the images are raw polar map, defect extent, and defect severity given by the Emory Cardiac Toolbox and its attenuation correction normal file.

Figure 6 shows the images of a normal subject. Bayesian AC produced images with less defect extent and severity, more similar to the AC normal database of the Emory Cardiac Toolbox than FBP AC.

## 4. DISCUSSION

This paper presents a transmission reconstruction technique, ABITR, for SPECT/slow-rotation low-output CT systems. Since the Hawkeye CT scanners use low-dose X-ray tubes, a typical transmission CT scan takes around 6 minutes to acquire 25 1-cm-thick slices. This slow rotation achieves good coregistration between the emission and transmission images; however, patient respiratory motion during the CT acquisition can result in inconsistency in the CT sinogram, and thus can create streak artifacts as we generally see in clinical practice. Due to the uncertainty in patient respiratory motion, it is difficult to manage the motion during the CT acquisition and to estimate where the streak artifacts will be present and how much they will impact AC. Nevertheless, the attenuation map with streak artifacts cutting through the myocardium may be of concern for clinicians and leads to questions regarding the possible impact of such attenuation maps on the quality of resulting AC images. From the patient studies presented in this paper, the ABITR technique showed to improve the quality of the slow-rotation low-output CT attenuation maps by eliminating the streak artifacts. It redistributed the pixel values in the attenuation maps to improve the soft-tissue uniformity while keeping the line integrals consistent to the data through iterative process. Compared to the FBP AC, ABITR AC yielded more uniform short-axis images for normal patient studies. In the phantom studies presented in this paper, ABITR and FBP yielded similar attenuation maps and AC images. This similarity indicated that ABITR did not create new artifacts in the attenuation map that can significantly impact AC. With phantom and patient studies, it is supported that ABITR can enhance the performance of the SPECT/slow-rotation low-output CT systems in AC in clinical MPI.

The original CT projections were in fan-beam geometry and had higher resolution (1-2 mm per pixel). It was rebinned to parallel-beam geometry with lower resolution (>6 mm per pixel). The FBP reconstruction was done before the rebinning, whereas the ABITR and OSEM reconstructions were done after the rebinning. In other words, the ABITR AC had three differences from the FBP AC: (a) Bayesian processing, (b) iterative reconstruction; and (c) image resolution (FBP was used before rebinning, whereas ABITR was used after rebinning). It has been shown in Table 2 that there were no significant differences between the OSEM AC and FBP AC studies, indicating that reduction of the transmission image resolution by rebinning did not help much in improving the attenuation-corrected images. In Figure 5, the OSEM map appears smoother than the FBP attenuation map but less smooth than the ABITR attenuation map. It must be noted that the Bayesian processing (assigning similar attenuation coefficients for the tissue region) does have a smoothing effect on the image, but the smoothing is different from the smoothing we generally use to reduce image noise. There is actually very little noise in the CT transmission data. In summary, among the three differences between ABITR AC and FBP AC, the Bayesian processing was shown to be the major contributor in improving the quality of the attenuation map.

The major limitation of this study is the small number of patient studies. With the small sample size, it only showed the statistical trend but did not reach the statistical significance (*P* < .05). Another limitation of this study is that no abnormal patients were included in the preliminary evaluation of the ABITR AC. A gold standard that can accurately measure defect extent and severity has not yet been established for comparison between the FBP AC and ABITR AC in abnormal patients. Nevertheless, the streak artifacts are more likely to create artifactual defects in the AC images of normal studies rather than to artifactually enhance the uniformity of the AC images on abnormal studies. The ABITR technique needs to be prospectively validated with a statistically sufficient sample size and with normal and abnormal patients and before it is implemented for clinical use. In addition, convergence test of the ABITR algorithm has not yet performed and a preset iteration number (30) was used in this study. It has been shown that iterative reconstruction of emission data converges around 20–50 iterations and then starts diverging when there is random noise in the emission data [21]. Since the transmission CT data has very little random noise, the ABITR algorithm is expected to converge quickly and to have very little divergence issues.

## 5. CONCLUSION

ABITR can remove the streak artifacts in the FBP attenuation maps caused by inconsistencies in the slow-rotation low-output CT sinogram such as those caused by patient respiratory motion during the acquisition. The improved quality of the ABITR attenuation map can yield more uniform attenuation-corrected images for normal subjects. ABITR can enhance the performance of SPECT/slow-rotation low-output CT systems in AC of clinical MPI. Prospective validation of this technique will be performed before the method is implemented for clinical use.

## Figures and Tables

**Table 1:** Short-axis uniformity and defect contrast analyses of the phantom studies.

Uniformity (%)	ABITR AC		FBP AC	

	Apical	Basal	Apical	Basal

Range	7.9–10.9	13.3–16.1	7.3–10.7	12.0–15.9
Mean	9.1	14.7	8.7	13.8
Std.	1.5	1.4	1.4	1.9
Mean Dif.*	0.4	0.9	—	—
Std. Dif.^§^	0.8	0.7	—	—
*P* ^†^	.3888	.0857	—	—

Contrast (%)	Anterior	Inferior	Anterior	Inferior

Range	50.0–56.7	47.3–49.7	49.1–56.0	46.7–48.5
Mean	53.1	48.3	53.5	47.5
Std	2.8	10.4	3.0	9.2
Mean Dif	−0.4	0.8	—	—
Std Dif	1.4	1.6	—	—
*P*	.5864	.3732	—	—

* The mean differences between the ABITR AC and FBP AC studies.

^§^ The standard deviation of the mean differences.

^†^ The *P* values were given by comparison between the ABITR AC and FBP AC studies using the paired *t*-test (*N* = 4). All of the *P* values in this table were greater than .05, indicating that there was no statistically significant difference between the ABITR AC and FBP AC images for the phantom studies.

**Table 2:** Short-axis uniformity analysis of the patient studies.

Uniformity (%)	ABITR AC			OSEM AC			FBP AC		

	Apical	Middle	Basal	Apical	Middle	Basal	Apical	Middle	Basal

Max.	9.2	8.1	21.1	9.0	8.2	22.4	8.8	7.9	22.0
Min.	4.4	5.2	12.7	4.8	5.3	13.7	5.0	5.9	12.9
Mean	7.2	6.6	15.8	7.3	7.0	16.5	7.1	7.1	16.3
Std.	2.0	1.2	3.7	1.8	1.3	4.0	1.6	1.0	4.1
Mean Dif.*	0.11	−0.43	−0.48	0.14	−0.16	0.21	—	—	—
Std. Dif.^§^	0.50	0.45	0.31	0.39	0.37	0.83	—	—	—
*P* ^†^	.6771	.1510	.0547	.5327	.4536	.6480	—	—	—

* The mean differences between the ABITR AC and FBP AC studies and between the OSEM AC and FBP AC studies.

^§^ The standard deviation of the mean differences.

^†^ The *P* values were given by comparison between the ABITR AC and FBP AC studies and between the OSEM AC and FBP AC studies using the paired *t*-test (*N* = 4). The *P* value of .0547 for the basal uniformities showed that the ABITR AC images had barely significantly better short-axis uniformity at the basal regions for the patient studies. No significant differences were obtained between the OSEM studies and FBP AC studies.
